# Supplementary material for: Identification of photoperiod-induced specific miRNAs in the adrenal glands of Sunite sheep (Ovis aries)
Source: Front Vet Sci. 2022 Jul 22;9:888207. doi: 10.3389/fvets.2022.888207 (PMC9354845; doi:10.3389/fvets.2022.888207)
Supplement: Supplementary file 1 [file Data_Sheet_1.ZIP › Supplementary file/miRDeep2 file.zip!/miRDeep2 file/Know_Structure/SP42A1/oar-mir-323a.pdf]

miRBase precursor : oar-mir-323a
Total read count : 1184
oar-miR-323a-5p read count : 4
oar-miR-323a-3p read count : 1180
remaining reads : 0

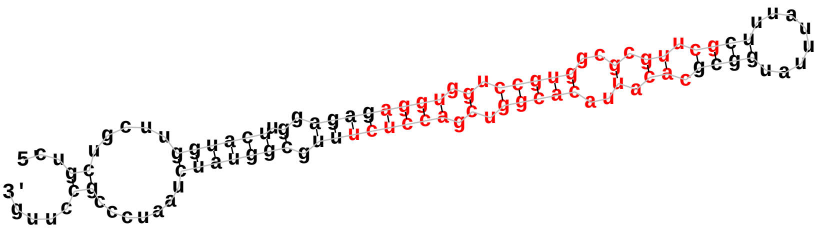

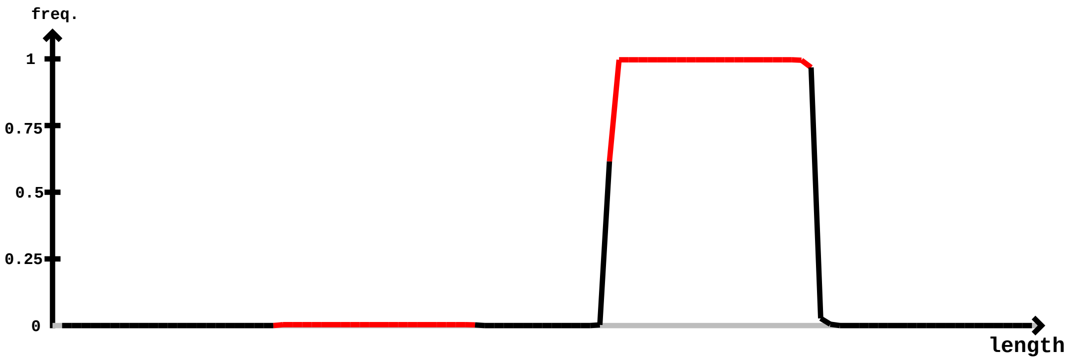


```
                     oar-miR-323a-5p
                                              oar-miR-323a-3p
5'- cugcugcuugguacuuggagagagguggucc guggcgcguucgcuuuauuuauggcgcacauuacacggucgaccucuuugcgguaucuaaucccgccuug  -3'   exp
    .........((((((.(.((((((((.(.(((((..(.((.((((........)))).)).)..))))).).)))))))).))))))).............   reads   mm   sample
    ......................agguggucguggcgcguuc...........................................................   1       0    seq
    ......................agguggucguggcgcguucg..........................................................   3       0    seq
    .......................................................cgcacauuacacggucgaccucu.......................   3       0    seq
    ........................................................gcacauuacacggucgaccu.........................   2       0    seq
    ........................................................gcacauuacacggucgaccuc........................   20      0    seq
    ........................................................gcacauuacacggucgaccucu.......................   690     0    seq
    ........................................................gcacauuacacggucgaccucuu......................   12      0    seq
    ........................................................gcacauuacacggucgaccucuuu.....................   2       0    seq
    .........................................................cacauuacacggucgaccuc........................   12      0    seq
    .........................................................cacauuacacggucgaccucu.......................   421     0    seq
    .........................................................cacauuacacggucgaccucuu......................   14      0    seq
    .........................................................cacauuacacggucgaccucuuu.....................   4       0    seq
```
